# Optimizing Rheology and Structure of Silver Pastes for Screen-Printed Silicon Solar Cells

**DOI:** 10.3390/ma19050918

**Published:** 2026-02-27

**Authors:** Baisen Hou, Zhiqiang Xia, Zhen Pang, Xinyu Zhou, Zhuo Qian, Wei Li, Mengyao Chai, Jiantao Yin, Junpeng Li, Xianglei Yu, Guoyou Gan

**Affiliations:** 1 Faculty of Material Science and Engineering, Kunming University of Science and Technology, Kunming 650093, China; houbaisen@stu.kust.edu.cn (B.H.); 20242130050@stu.kust.edu.cn (Z.X.); pangzhen@stu.kust.edu.cn (Z.P.); zhouxinyu1@stu.kust.edu.cn (X.Z.); 20210067@kust.edu.cn (Z.Q.); 202211605123@stu.kust.edu.cn (M.C.); 202211605113@stu.kust.edu.cn (J.Y.); 2 Sino-Platinum Electronic Materials (Yunnan) Co., Ltd., Kunming 650503, China; li_gzu@126.com; 3 Engineering Training Center, Kunming University of Science and Technology, Kunming 650093, China

**Keywords:** silver paste, organic vehicle, rheological properties, thixotropy, height-to-width ratio

## Abstract

Solar energy, as a clean and renewable resource, plays a pivotal role in advancing sustainable energy technologies. The efficiency of front-side silver paste is critical for the photovoltaic performance of Tunnel Oxide Passivated Contact (TOPCon) solar cells. In this study, we comprehensively investigated how the composition of organic vehicles in conductive pastes influences both printing rheological properties and electrical performance. Through rheological characterization, contact angle measurements, and Three-Interval Thixotropy Tests (3ITT), we examined the effects of varying solvent, binder, and thixotropic agent ratios on paste properties. The optimized formulation—a solvent mixture of lauryl alcohol ester (TE), butyl carbitol (DGME), butyl carbitol acetate (BCA), and dibutyl phthalate (DBP) in a 3:4:2:1 ratio, with ethyl cellulose (EC) STD10 as the binder and a polyamide wax (PAW)–hydrogenated castor oil (HCO) thixotropic agent at a 3:1 mass ratio—demonstrated superior viscosity control and rapid structural recovery. Printed grid lines achieved a height-to-width ratio (H/W) of 0.35 and a sheet resistance (Rs) of 1.43 Ω/□. These findings reveal direct relationships between organic vehicle composition, paste rheology, and functional performance, providing practical guidance for the design and optimization of high-performance conductive pastes for c-Si solar cells. This work establishes a foundation for improving both the efficiency and reliability of next-generation silver paste formulations in photovoltaic applications.

## 1. Introduction

The instability of the global energy supply, driven by both the depletion of fossil fuels and rising geopolitical tensions, has accelerated efforts worldwide to shift toward sustainable alternative [1]. Among the various renewable technologies, photovoltaic (PV) systems have emerged as a promising solution due to their operational safety, low environmental impact, and scalability [2]. Tunnel Oxide Passivated Contact (TOPCon) solar cells, which currently dominate the PV market, continue to enhance their cost–performance ratio through improvements in device efficiency and manufacturing techniques [3,4,5,6]. The efficiency of photovoltaic panels is influenced not only by cell architecture but also by front-side metallization, which directly affects series resistance and optical shading losses. In high-efficiency silicon solar cells, precise control over silver grid-line geometry and electrical conductivity is therefore essential for achieving improved power conversion efficiency. In addition to photovoltaic applications, conductive electronic pastes have attracted increasing attention for their critical role in high-performance sensors, where stable electrical conductivity and reliable signal transmission are essential for accurate sensing and long-term operation [7,8,9].

The performance of printed electrodes is closely linked to the physicochemical properties of the silver paste. As a complex multicomponent dispersion, the paste’s rheology governs its ability to flow through the screen, retain its shape after deposition, and form a dense, conductive structure upon sintering [10,11,12]. Central to these properties is the organic vehicle, which regulates particle interactions, controls shear-induced structural breakdown, and influences the recovery of the internal network after shear removal [13,14]. In particular, oil-based components in the organic vehicle play a crucial role in the formation of thixotropic systems by providing a continuous medium that enables sufficient flow under shear and rapid structural recovery after shear cessation, which is essential for stable paste formulation and reliable printing performance. Studies by Gao [13] et al. have shown that variations in solvent composition, binder type, and additive content can markedly affect viscosity behavior, printing fidelity, and final electrode morphology [15,16,17,18].

Although considerable progress has been made, our understanding of how vehicle formulations influence the moment-to-moment reconstruction of printed gridlines is still limited. Prior studies have largely emphasized steady shear properties, while the short-lived structural responses that occur during printing—measurable through the Three-Interval Thixotropy Test (3ITT)—have been explored far less thoroughly. For this reason, the present study centers on the rheological behavior of the organic vehicle [19,20], seeking a system that can flow readily when subjected to shear but regain its structure quickly after shear ceases, so that fine silver gridlines with high aspect ratios and low electrical resistance can be reliably produced [19,20,21].

In this study, we methodically examine how the composition of organic vehicles—including mixed solvent ratios, binder formulations, and thixotropic agents—affects the rheology and printing behavior of silver pastes for TOPCon solar cell metallization. By combining steady-shear measurements, 3ITT recovery analysis, screen-printing trials, and three-dimensional morphological evaluation, we establish direct correlations between vehicle-dependent rheological behavior and resulting grid-line geometry [21]. Our results provide clear guidelines linking formulation parameters to flow behavior, structural reconstruction, and the electrical performance of screen-printed silver electrodes, offering practical insights for the optimization of metallization pastes used in high-efficiency silicon solar cells [22,23,24].

## 2. Materials and Methods

### 2.1. Experimental Materials

TOPCon silicon wafers with dimensions of 182 mm × 182 mm were used as substrates for screen printing. The organic vehicles were prepared using the following chemical reagents: ethyl cellulose(EC) (Dow Chemical Company, Midland, MI, USA), butyl carbitol (DGME) (98%, Aladdin, Shanghai, China), butyl carbitol acetate(BCA) (98%, Aladdin, Shanghai, China), lauryl alcohol ester(TE) (Macklin, Shanghai, China), dibutyl phthalate(DBP) (Macklin, Shanghai, China), coupling agent KH-570 (Shuguang, Nanjing, China), lecithin (Macklin, Shanghai, China), polyamide wax(PAW) (Macklin, Shanghai, China), hydrogenated castor oil(HCO) (Aladdin, Shanghai, China), and Span 85 (Xingfu Fine Chemicals, Tianjin, China). The glass powders were synthesized from Bi_2_O_3_ (99.9%, Aladdin, Shanghai, China), TeO_2_ (99.9%, Kevtech, Beijing, China), B_2_O_3_ (98%, Aladdin, Shanghai, China), Al_2_O_3_ (AR, Sinopharm Chemical Reagent Co., Ltd., Shanghai, China), ZnO (AR, Sinopharm Chemical Reagent Co., Ltd., Shanghai, China), SiO_2_ (98.5%, Sinopharm Chemical Reagent Co., Ltd., Shanghai, China), Li_2_O (99.9%, Aladdin, Shanghai, China). Silver powders used for the conductive paste were obtained from Sino-Platinum Metals Co., Ltd., Kunming, China. Screen-printing meshes with an average aperture of approximately 1 mm were sourced from Kunshan Silk Screen Products Co., Ltd., Kunshan, China.

### 2.2. Preparation of Organic Vehicles

**Figure 1:**
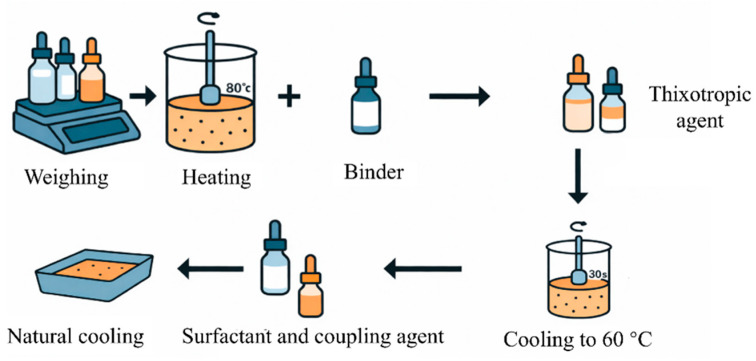
Preparation process of the organic vehicle.

As shown in Figure 1, organic vehicles were formulated based on previous studies on silver paste rheology and organic vehicles. The preparation procedure followed commonly adopted methods reported in the literature for screen-printing silver paste organic vehicles, with controlled processing parameters to ensure reproducibility. Solvents, binders, thixotropic agents, surfactants, and coupling agents were accurately weighed. Solvents were first added to a beaker on a heated magnetic stirrer (Yuezhong Instrument Equipment Co., Ltd., Shanghai, China, 300 rpm, 80 °C) and activated. The binder was then incorporated until fully dissolved. The mixture was cooled to 60 °C and stirred for 30 s before sequentially adding thixotropic agents and surfactants, as addition order critically affects the organic vehicle’s performance. The resulting organic vehicles were obtained after natural cooling for 3 h. In this study, the system under consideration is a screen-printing silver paste composed of silver powder, glass frit, and an organic vehicle, which serves as the primary rheology-controlling component through the formation of a shear-sensitive internal network.

### 2.3. Preparation of Glass Powder

The oxides were weighed according to composition: Bi_2_O_3_ (45%), TeO_2_ (20%), B_2_O_3_ (20%), SiO_2_ (9%), Al_2_O_3_ (3%), ZnO (2%) and Li_2_O (1%) mixed uniformly in a mortar. The mixture was transferred to a crucible and sintered at 1000 °C for 30 min, followed by water quenching. The quenched samples were ground, planetary ball-milled with agate balls and ethanol (mass ratio 3:2:1) for 24 h at 300 rpm, centrifuged, dried at 60 °C for 10 h, crushed, and sieved through a 400-mesh screen to obtain the final glass powder.

### 2.4. Preparation of Silver Paste

**Figure 2:**
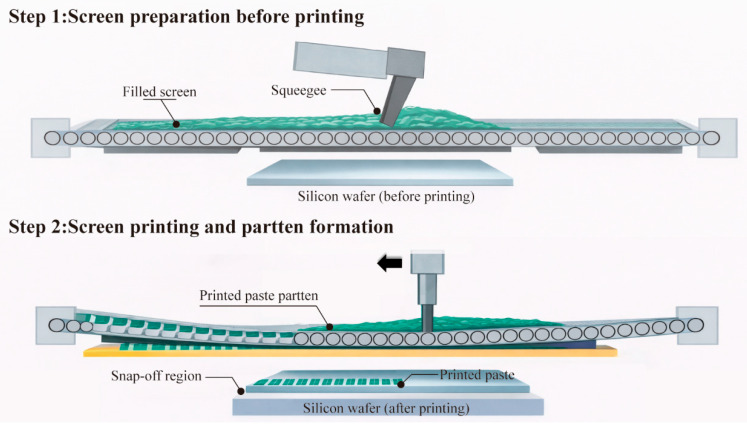
Schematic illustration of the screen-printing process (arrow denotes the squeegee movement direction).

Silver paste was prepared by mixing silver powder (88%), organic vehicle (10%), and glass powder (2%) in an agate mortar. The mixture was processed using a three-roll mill (front gap 3 mm, rear gap 5 mm) to ensure uniform dispersion, following established procedures. The paste was screen-printed onto Topcon wafers, as schematically shown in Figure 2, dried at 160 °C for 20 min, and sintered at 820 °C for 5 min in a muffle furnace to produce solar cell electrodes. In this study, the system under consideration is a screen-printing silver paste composed of silver powder, glass frit, and an organic vehicle, which serves as the primary rheology-controlling component through the formation of a shear-sensitive internal network.

### 2.5. Characterizations

Rheological properties of the organic vehicles and silver pastes, including viscosity and thixotropy, were characterized using a rotational rheometer (RST-SST, Brookfield, Middleboro, MA, USA) to examine their flow behavior under shear conditions relevant to screen printing. Structural recovery was evaluated by three-interval thixotropy tests (3ITT), in which a controlled high-shear step was applied to disrupt the internal structure, followed by monitoring the viscosity evolution after shear removal to assess the reversibility of the structural response. The wetting behavior of the organic vehicle on silicon substrates, glass powder, and silver powder was examined using a contact angle goniometer (OCA 15EC, DataPhysics, Filderstadt, Germany), providing insight into the interfacial compatibility between the liquid phase and different solid components. The microstructure and particle distribution of the sintered silver grid lines were observed by optical microscopy (Olympus, Tokyo, Japan) to assess line continuity and surface uniformity. Three-dimensional surface morphology and cross-sectional profiles of the printed grid lines were measured using an optical profilometer (MarSurf LD130, Mahr, Göttingen, Germany), from which the height-to-width ratio (H/W) was determined. The sheet resistance (Rs) of the sintered films was measured using a standard four-point probe system (ST-2258C, Suzhou Jingge Electronic, Suzhou, China) to evaluate the electrical performance of the printed electrodes. Although bidirectional shear rate sweep tests were not explicitly presented, the three-interval thixotropy test (3ITT) employed in this study provides an equivalent assessment of structural breakdown and recovery under shear, which is physically analogous to the hysteresis behavior observed in increasing–decreasing shear rate flow curves reported in the literature.

## 3. Results and Discussion

### 3.1. The Selection of Organic Solvents

To ensure uniform evaporation during screen printing, drying, and sintering, as well as proper wetting on all component surfaces, mixed organic solvents were employed. It is well established that the rheological properties of silver pastes strongly influence their printing performance [13]. In this study, twelve solvent formulations with varying ratios were designed using an orthogonal approach, as summarized in Table 1.

The four selected solvents included TE, DGME, BCA, and DBP. After thorough stirring at room temperature, 5 g of each formulation was placed into a heat-resistant beaker and subjected to stepwise heating in a hot-air oven. The samples were heated sequentially from 60 to 220 °C, holding each temperature for 5 min. Mass measurements were recorded at each interval to determine volatilization loss according to Equation (1).

(1) $$\mathrm{Volatility}\;(\%)=\frac{m_{0}-m_{t}}{m_{0}}\times 100\%\;$$

$m_{0}:Initial\;mass\;(g),$ $m_{t}:Mass\;at\;time\;t\;(g),$ Volatility: Volatilization rate (%).

**Figure 3:**
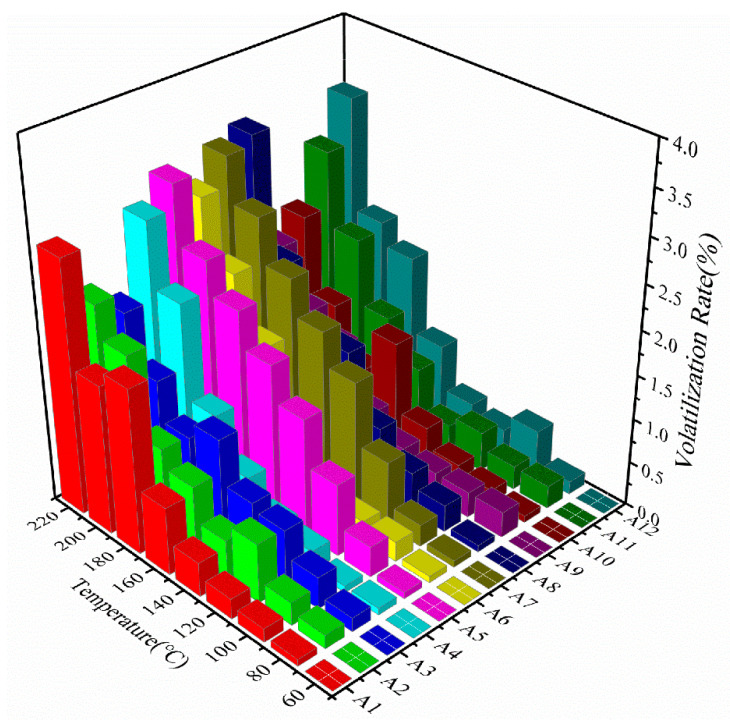
Evaporation profiles of twelve mixed organic solvent formulations.

Previous studies have demonstrated that organic solvents play a pivotal role in controlling both volatilization behavior and the rheological performance of silver pastes [25]. The formulations with the most stable evaporation profiles were identified based on smooth, nearly linear mass-loss curves, showing no abrupt transitions over the 60–220 °C range. As illustrated in Figure 3, formulations A5, A7, and A9 maintained relatively uniform volatilization throughout the entire temperature interval. It is widely recognized that the composition of mixed solvent systems significantly affects the rheological behavior of conductive pastes [13,25].

**Figure 4:**
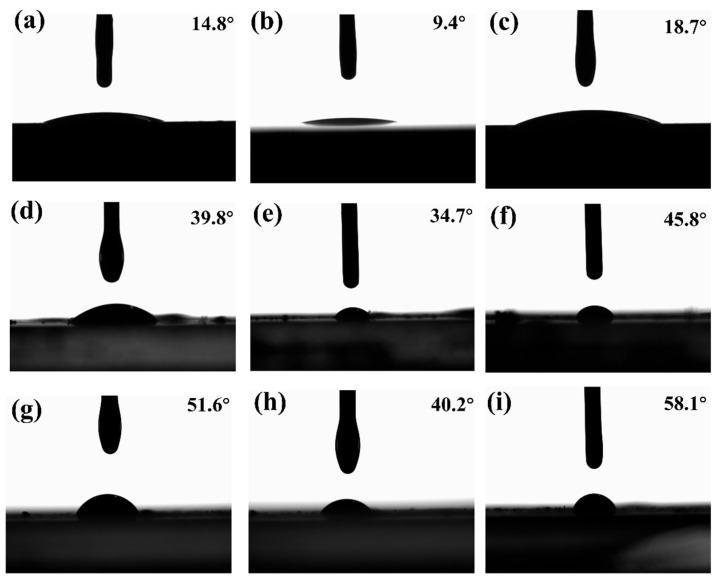
Contact angles of selected solvent formulations on different substrates: (**a**,**d**,**g**) A5; (**b**,**e**,**h**) A7; (**c**,**f**,**i**) A9, on silicon wafer, glass powder, and silver powder, respectively.

To further evaluate substrate compatibility, contact angle measurements were performed for A5, A7 and A9 on silicon wafers, glass powder, and compacted silver powder. A small droplet of each solvent was applied to the substrate surface, and the contact angles were recorded over the first 0–5 s and at equilibrium using a goniometer, as illustrated in Figure 4. A5 showed contact angles of 14.8°, 39.8° and 51.6° on silicon, glass, and silver, respectively, while A9 exhibited 18.7°, 45.8°, and 58.1°. Among the tested formulations, A7 demonstrated the best wetting behavior, with equilibrium angles of 9.4°, 34.7°, and 40.2° on the three substrates. Considering both volatilization stability and wetting performance, A7 was selected as the optimal solvent for screen printing, drying, and sintering. These results highlight the critical influence of solvent choice on substrate wetting and provide guidance for evaluating the compatibility of organic solvents with different materials [26].

### 3.2. The Selection of Binder

Binder selection was performed using an expert-guided, stepwise experimental screening approach. Based on prior experience in silver paste formulation and reported rheological requirements for screen printing, twelve organic vehicle formulations (M1–M12) with different binder ratios were designed. The detailed compositions of these formulations are listed in Table 2. Ethyl cellulose with different viscosity grades (STD7, STD10, STD20, and STD45), corresponding to different molecular weight ranges, was used as the polymer binder to investigate the influence of binder molecular characteristics on rheological behavior. In all cases, viscosity decreased with increasing shear rate, exhibiting typical shear-thinning behavior characteristic of non-Newtonian fluids [27]. Such behavior enables the paste to flow smoothly during printing while maintaining structural integrity at rest, fulfilling essential requirements for front-side silver paste processability [14,15,16,27].

**Figure 5:**
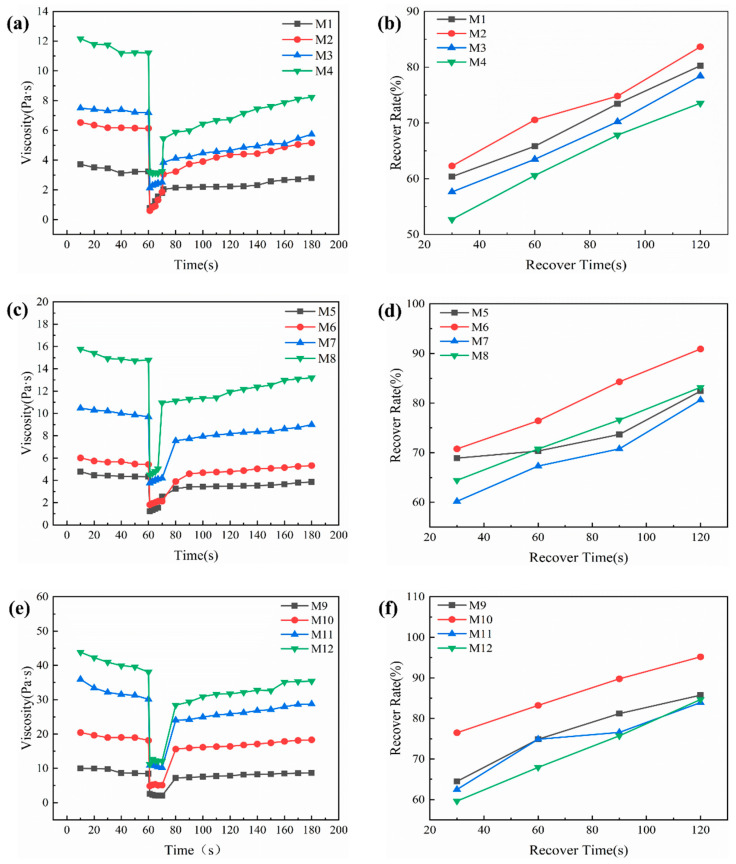
(**a**,**c**,**e**) Three-Interval Thixotropy Test (3ITT) curves of M1–M12; (**b**,**d**,**f**) corresponding structural recovery rates of M1–M12.

To evaluate the structural breakdown and recovery of the silver paste under printing conditions, a Three-Interval Thixotropy Test (3ITT) was performed. The resulting shear response curves are presented in Figure 5a,c,e. The test included three sequential stages to mimic the shear conditions encountered during printing: an initial low shear rate of 1 s^−1^ representing the resting state; a high shear rate of 80 s^−1^ to simulate network disruption, during which viscosity dropped sharply; and a return to 1 s^−1^ to monitor structural recovery after shear cessation [14,27]. Structural recovery rates, calculated according to Equation (2), are shown in Figure 5b,d,f. Among the tested binders, STD10 consistently achieved the highest recovery efficiency [25]. This is attributed to its intermediate molecular weight, which provides an optimal balance between flowability under shear and rapid network reconstruction afterward. In comparison, STD7, with a lower molecular weight, exhibits low viscosity and flows readily but shows weak thixotropic behavior, limiting structural recovery. Conversely, STD20 and STD45, with higher molecular weights, form stable three-dimensional networks that enhance thixotropy and suspension stability, but their reduced flowability can impair printing uniformity. Considering these characteristics, STD10 was chosen as the primary binder for all subsequent experiments [28,29].

(2) $$R\left(\%\right)=\frac{\eta_{3}-\eta_{2}}{\eta_{1}-\eta_{2}}\times 100\;$$

(%): Structural recovery rate, expressed as a percentage (%),

$\eta_{1}$: Viscosity of the paste during the initial low-shear stage (Pa·s),

$\eta_{2}$: Viscosity of the paste during the high-shear stage (Pa·s),

$\eta_{3}$: Viscosity of the paste during the recovery stage after shear cessation (Pa·s).

### 3.3. Selection of Thixotropic Agents

To enhance the rheological performance of the silver paste, particularly its flowability under high shear during screen printing and its ability to rapidly recover structure after shear removal, STD10 was selected as the binder, aiming to achieve a balanced combination of sufficient flowability under high shear during printing and rapid structural recovery after shear removal., and a mixed solvent composed of TE, DGME, BCA, and DBP in a mass ratio of 3:4:2:1 was employed as the dispersing medium to formulate the organic vehicle system. Before discussing the thixotropic recovery behavior, it should be noted that all silver paste formulations exhibit a typical shear-thinning flow behavior, as evidenced by the monotonic decrease in viscosity with increasing shear rate. Such flow characteristics are consistent with flow curves commonly reported for screen-printing silver pastes and provide the basis for subsequent thixotropic analysis. The influence of HCO and PAW on the thixotropic behavior of the vehicle was systematically investigated, and five different formulation schemes were designed, as listed in Table 3, to optimize the thixotropic response. The primary objective of this study was to improve the printability and structural stability of the silver paste, thereby providing a foundation for subsequent process performance optimization [15].

**Figure 6:**
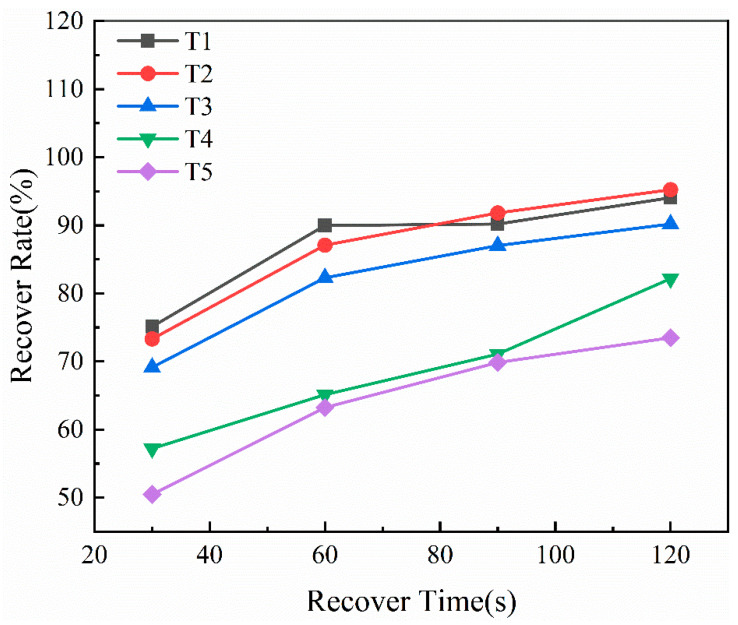
Structural recovery rate of organic vehicle systems T1–T5.

The impact of different thixotropic-agent ratios on the structural recovery of the organic vehicle was investigated through recovery-rate analysis [26]. As shown in Figure 6, all formulations exhibited a gradual increase in recovery with resting time (30–120 s). T1, T2, and T3 demonstrated faster recovery, exceeding 80% at 90 s and nearing 90% at 120 s, while T4 and T5 recovered more slowly, indicating a weaker network reconstruction ability. These results highlight the importance of thixotropic-agent composition in controlling the reversible network and, consequently, the rheological stability of the paste [13,26].

**Figure 7:**
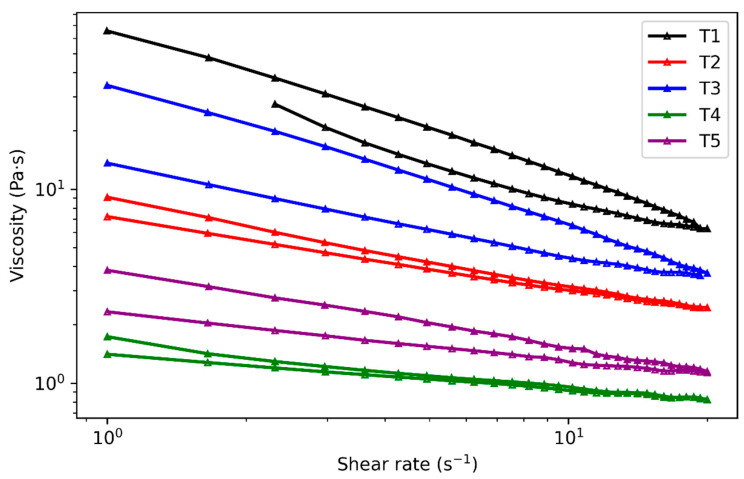
Thixotropic hysteresis loops of organic vehicle systems T1–T5.

To further elucidate the reversible structural behavior of the organic vehicle under shear, thixotropic hysteresis loops were characterized by viscosity–shear rate cyclic scanning, as shown in Figure 7. The shear rate was cycled from 1 s^−1^ to 20 s^−1^ and back to 1 s^−1^ to simulate the typical shear conditions experienced during screen printing and to capture both shear-induced structural breakdown and post-shear reconstruction. All measurements were conducted at 25 °C under controlled rotational mode.

Based on the combined analysis of structural recovery rates and thixotropic hysteresis loops, the different thixotropic systems exhibit pronounced differences in shear response and structural reconstruction behavior. According to the results, the five systems can be classified into three representative behavior modes. The T1 system, dominated by polyamide wax (PAW), shows strong structural support but a relatively limited recovery rate after shear. In contrast, the T5 system, primarily based on hydrogenated castor oil (HCO), exhibits high flowability but insufficient structural reconstruction capability. The remaining PAW–HCO composite systems demonstrate a more balanced thixotropic response, achieving a compromise between high-shear flowability and post-shear structural recovery. Furthermore, analysis of the low-shear viscosity characteristics reveals that the T4 and T5 systems possess relatively low overall viscosity, making it difficult to rapidly establish sufficient structural support after shear removal to suppress paste spreading. This behavior is unfavorable for maintaining morphological stability during subsequent printing processes.

Among the investigated systems, the T2 and T3 systems exhibit relatively moderate hysteresis loop areas, indicating a balanced combination of shear-induced flowability and post-shear structural recovery. Such thixotropic behavior is considered optimal for photovoltaic screen-printing applications, as it enables smooth paste transfer through the screen while effectively suppressing line spreading after printing. In contrast, the T1 system, characterized by a larger hysteresis loop and stronger structural rigidity, was retained as a representative high-structure system for subsequent comparative analysis.

### 3.4. Analysis of Leveling Behavior and Printed Silver Grid Morphology

The effect of different organic vehicle systems on the printability of silver paste was investigated by examining the surface morphology of printed and sintered silver grid lines via optical microscopy [19,20], with leveling behavior assessed based on the formulations summarized in Table 4. Generally, excellent leveling is indicated by straight edges, continuous lines, and well-defined interfaces, reflecting good morphological stability and suitable thixotropic recovery of the paste after printing [19,20,26].

**Figure 8:**
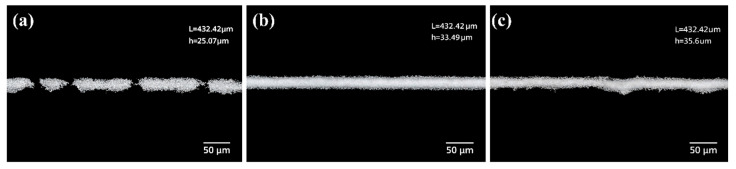
Optical microscopy images of silver grid lines: (**a**) S1; (**b**) S2; (**c**) S3.

As shown in Figure 8, pronounced differences in the printed silver grid morphologies are observed among the pastes formulated with different thixotropic systems, highlighting the critical role of post-shear structural reconstruction in determining printing fidelity.

For the S1 system containing only polyamide wax (PAW), the printed grid lines exhibit noticeable discontinuities and local material accumulation. Although the high viscosity provides strong structural support, the overly compact internal network cannot be sufficiently disrupted under the shear imposed by the squeegee, resulting in incomplete mesh release and non-uniform line formation. Similar phenomena have been reported in previous studies, where excessive structural strength limits effective paste transfer during screen printing and leads to morphological defects. In contrast, the S2 system with a PAW-to-HCO ratio of 3:1 produces continuous silver grid lines with uniform widths, well-defined edges, and relatively consistent line heights. This morphology reflects a balanced combination of high-shear flowability and rapid structural reconstruction after shear removal, which effectively suppresses post-printing spreading. Such a compromise between flow behavior and shape stability has been widely recognized as a key requirement for high-quality printed silver grids. For the S3 system with a higher HCO content, the reduced viscosity facilitates paste transfer through the screen; however, the insufficient post-shear structural recovery leads to blurred edges, local collapse, and increased width fluctuations along the grid lines. This behavior is consistent with previous reports indicating that inadequate structural reconstruction after shear cessation can result in morphological instability during the resting stage.

Overall, the optical microscopy results confirm that the printed morphology of silver grid lines is not governed solely by high-shear flowability, but rather by the synergistic balance between flow behavior under shear and structural reconstruction after shear cessation. An appropriate combination of PAW and HCO is therefore critical for achieving stable and high-quality printed silver grid lines, in agreement with established rheology–morphology correlations reported in the literature [30].

### 3.5. Correlation Analysis Between Silver Grid Line H/W and Rs

#### 3.5.1. Geometrical Morphology Analysis (Aspect Ratio)

**Figure 9:**
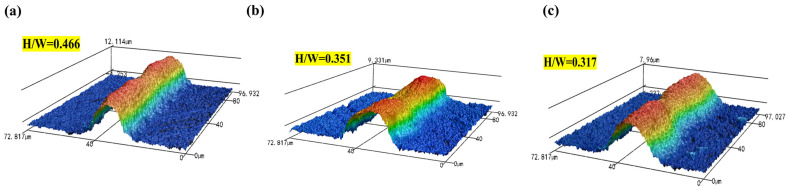
3D microscopic image of silver after screen printing: (**a**) S1; (**b**) S2; (**c**) S3.

The cross-sectional geometries of the silver grid lines in samples S1, S2, and S3 were characterized by optical profilometry, from which the height-to-width ratio (H/W) was determined [20,21,24,31,32]. As shown in Figure 9, which corresponds to the silver grid morphologies of samples S1, S2, and S3, respectively, all samples exhibit continuous grid line structures, but noticeable differences in cross-sectional geometry were observed. The silver grid lines in sample S2 show a nearly trapezoidal cross-sectional profile with a flat top and clear edges, without any apparent collapse, yielding an H/W value of approximately 0.35. Owing to its relatively low H/W ratio, sample S3 is prone to lateral spreading of the silver grid lines, leading to reduced morphological stability, whereas sample S1 exhibits an excessively high H/W ratio and a steep cross-sectional profile, potentially increasing the risk of structural instability during drying and sintering, indicating slower structural recovery or excessive flow of the paste [14,25]. The conductive silver paste in the S2 system maintains sufficient flowability under high shear, enabling smooth paste transfer during screen printing, while rapidly reconstructing its three-dimensional network after shear removal to effectively stabilize the grid-line morphology. The moderate H/W ratio suppresses excessive lateral spreading while avoiding excessive vertical build-up, thereby enhancing geometric stability [14,33,34]. The moderate H/W ratio enhances both series resistance reduction and shading loss mitigation, demonstrating superior shape fidelity and geometric stability for this formulation [18,19,21,22,35].

#### 3.5.2. Electrical Performance Measurement (Rs)

**Figure 10:**
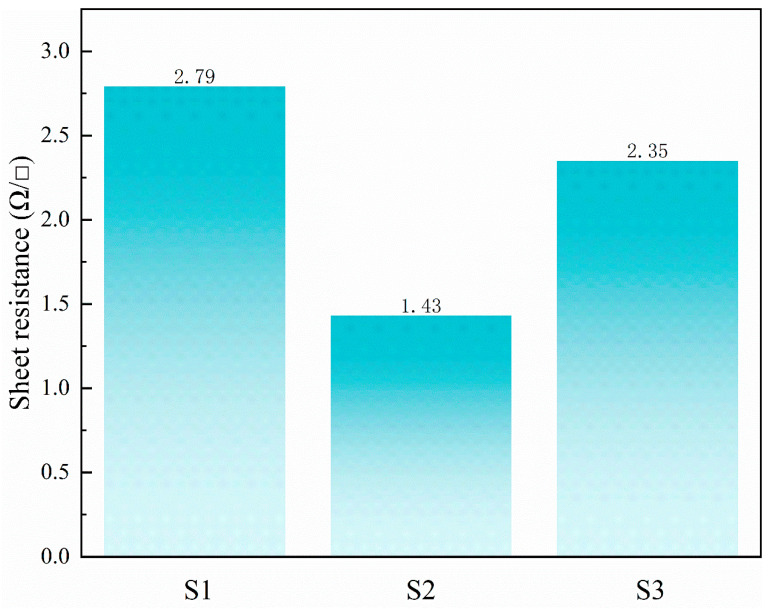
Sheet resistance (R_s_) of printed silver grid lines for samples S1–S3.

To further assess the electrical conductivity of the silver grid lines, the Rs of each sample was determined using a standard four-point probe method [36,37]. The sheet resistance values of samples S1, S2, and S3 are shown in Figure 10. Among them, S2 exhibited the lowest sheet resistance (1.43 Ω/□), consistent with its superior geometric profile. The higher aspect ratio and smoother surface of S2 provide a larger effective conductive cross-section and a more continuous electron transport path, thereby reducing electron scattering and enhancing charge transfer efficiency. This behavior can be attributed to the synergistic effect of rheological recovery and microstructural stabilization of the printed grid lines. The optimized thixotropic response in S2 suppresses lateral spreading while maintaining sufficient vertical build-up, resulting in a denser and more continuous conductive pathway after sintering. Consequently, electron scattering at grain boundaries is reduced, leading to lower sheet resistance. These results further confirm that the electrical performance of screen-printed silver electrodes is strongly governed by microstructural characteristics, in agreement with previous studies [4,23,36,37].

In addition to reflecting improved microstructural connectivity, the reduced sheet resistance of S2 is also indicative of lower series resistance in the printed electrodes. This is beneficial for photovoltaic applications, as it can reduce resistive power losses during current collection and contribute to improved device efficiency. Moreover, the formation of dense and continuous conductive pathways is expected to enhance the current uniformity and electrical stability of the printed grid lines during operation.

## 4. Conclusions

This study systematically investigated how the composition of organic solvents, binder types, and thixotropic agent ratios influenced the rheological behavior, aspect ratio, and sheet resistance of conductive silver pastes.

1. The mixed solvent system A7 was identified as optimal, improving substrate wettability and adhesion while promoting uniform solvent evaporation after screen removal.
2. Incorporating STD10 as the binder provided strong flow under high shear and rapid structural recovery once shear was relieved, ensuring the printed lines retained their shape.
3. Under the optimized organic vehicle formulation, the S2 system with an HCO: Paw ratio of 3:1 exhibited a pronounced improvement in rheological behavior and printing stability. This enhancement originates from a reversible shear-induced internal network, which allows sufficient flow under high shear during printing and rapid structural recovery after shear removal. As a result, dense and continuous silver grid lines with enhanced structural integrity and sharper edges were formed, achieving the highest aspect ratio (0.35) and the lowest sheet resistance.
4. Overall, the synergistic interaction among organic solvents, binders, and thixotropic agents plays a crucial role in tailoring paste performance. Proper formulation balance enables both high printability and structural–electrical stability, providing a practical strategy and experimental foundation for developing high-precision printed electronic materials.
5. From an application perspective, the proposed formulation strategy shows favorable cost implications for industrial screen-printing. The optimization is achieved by adjusting the organic vehicle (10 wt%), while the silver powder content, which dominates material cost, remains unchanged at 88 wt%. This indicates that improved printability and electrical performance can be achieved without a noticeable increase in production cost, offering a cost-neutral and scalable solution for photovoltaic metallization.

## Figures and Tables

**Table 1:** Compositions of the twelve mixed organic solvent formulations.

Sample	TE	DGME	BCA	DBP
A1	1	1	1	1
A2	1	2	2	2
A3	1	3	3	3
A4	2	1	2	3
A5	2	2	3	1
A6	2	3	1	2
A7	3	4	2	1
A8	3	2	1	3
A9	3	3	2	1
A10	4	2	1	3
A11	4	1	3	2
A12	4	1	3	1

**Table 2:** Detailed compositions of M1–M12 with different binder ratios.

Sample	Solvent	STD7	STD10	STD20	STD45
M1	A7	14%	0	0	0
M2	A7	0	14%	0	0
M3	A7	0	0	14%	0
M4	A7	0	0	0	14%
M5	A7	16%	0	0	0
M6	A7	0	16%	0	0
M7	A7	0	0	16%	0
M8	A7	0	0	0	16%
M9	A7	18%	0	0	0
M10	A7	0	18%	0	0
M11	A7	0	0	18%	0
M12	A7	0	0	0	18%

**Table 3:** Detailed composition of thixotropic agents in formulations T1–T5.

Sample	Solvent	Binder	Thixotropy
T1	A7	STD10	PAW
T2	A7	STD10	PAW: HCO = 3:1
T3	A7	STD10	PAW: HCO = 1:1
T4	A7	STD10	PAW: HCO = 1:3
T5	A7	STD10	HCO

**Table 4:** Detailed composition of Silver Paste in formulations S1–S3.

Sample	Silver Powder	Glass Powder	Organic Vehicle
S1	88%	2%	10% (T1)
S2	88%	2%	10% (T2)
S3	88%	2%	10% (T3)

## Data Availability

The original contributions presented in this study are included in the article. Further inquiries can be directed to the corresponding authors.
